# Increased Fluctuating Asymmetry in a Naturally Occurring Hybrid Zone between the Stick Insects *Bacillus Rossius Rossius* and *Bacillus Rossius Redtenbacheri*

**DOI:** 10.1673/031.010.14107

**Published:** 2010-09-10

**Authors:** Ditte Demontis, Cino Pertoldi, Marco Passamonti, Valerio Scali

**Affiliations:** ^1^ Department of Biological Sciences, Aarhus University, Ny Munkegade, Building 1540, 8000 Århus C, Denmark; ^2^ Department of Human Genetics, Aarhus University, Wilhelm Meyers Allé 4, 8000 Århus C, Denmark; ^3^ Dipartimento di Biologia Evoluzionistica Sperimentale, Universitá di Bologna, Via Selmi 3, 1-40126 Bologna, Italy; ^4^ Mammal Research Institute, Polish Academy of Sciences, Waszkiewicza 1c, 17-230 Białowieża, Poland

**Keywords:** genetic incompatibility, hybridization, microsatellites, phenotypic variance

## Abstract

The impact of interracial hybridization on fluctuating asymmetry (FA) and phenotypic variability (σ^2^p) in a presumed natural hybrid zone between the stick insects, *Bacillus rossius rossius* Rossi and *Bacillus rossius redtenbacheri* Nasceti & Bullini (Phasmatodea: Bacillidea), found on the Italian island Sardinia was investigated. The lengths of three bilateral traits and three unilateral traits were measured, and each individual was genotyped by five microsatellite loci. The genotypic data clearly confirmed the existence of the hybrid zone on Sardinia. A significantly increased FA was found in the hybrids when compared to both parental subspecies, which this study attributes to genetic incompatibilities in the hybrids. The increase in FA was not correlated with any increase in σ^2^P in the hybrids, which indicates that in this case σ^2^p and FA originate from separate processes.

## Introduction

In nature, many stable natural hybrid zones exist in which hybrid populations have persisted for centuries (Bella et al. 2007; Szymura and Barton 1986). In many cases hybridization has been an important part of evolution that promotes incorporation of new genes from one population to another and in several cases has been shown to be a step in the process leading to speciation (Templeton 1981; Mantovani et al. 1996; Parris et al. 2001). The stability of hybrid zones is very often attributed to a balance between dispersal of parental individuals into the zone and selection against hybrids (Barton and Hewitt 1985; Szymura and Barton 1986; Ostberg et al. 2004).

Hybridization may affect fitness of hybrid individuals, and several investigations have shown that the fitness of hybrids is often decreased dramatically. Furthermore, hybridization may affect the developmental instability (DI) of hybrids, which refers to an individual's inability to produce a specific phenotype under a given set of environmental conditions. A number of studies have shown DI to be increased in hybrids when compared to their parentals (e.g. Andersen et al. 2002, 2006; Garnier et al. 2006). One commonly used measure of DI is fluctuating asymmetry (FA), or the difference in value between paired bilateral traits, which is known to be elevated due to environmental stresses and genetic factors such as hybridization (Andersen et al. 2002, 2006, 2008; Kristensen et al. 2003, 2004; Røgilds et al. 2005; Pertoldi et al. 2006a; Petavy et al. 2006; Lens et al. 2000; Soderman et al. 2007; Krag et al. 2009). The reason for the increased FA in intraspecific hybrids has been attributed to a breakdown in co-adapted gene complexes (Andersen et al. 2002), whereas in interspecific hybrids increased FA has more often been attributed to genetic incompatibilities causing meiotic irregularities or physiological and developmental abnormalities (Andersen et al. 2006; Burke and Arnold 2001; Pialek et al. 2001; Rego et al. 2006; Kurbalija et al. 2010).

Under stressful conditions several investigations have found an increase in DI estimated as FA and the phenotypic variance (σ^2^p) (Imasheva and Bubliy 2003; Willmore et al. 2005, 2006; Pertoldi and Bach 2007). Even though this is a common observation, the underlying mechanisms that control σ^2^p and FA are still not clear (Pertoldi et al. 2001, 2003, 2006b). Waddington (1957) originally stated that σ^2^p and FA are controlled by separate processes, namely canalization and developmental stability, respectively. Canalization describes the buffering mechanisms that ensure similar development under different environmental influences, which, according to Waddington (1957), should be separated from the process responsible for the within-individual variability (DI) resulting in FA.

The question of whether canalization (σ^2^p) and DI (FA) are affected by different mechanisms has been doubted by several authors (Dworkin 2005; Willmore et al. 2006), and supported by others (Hoffmann and Woods 2001; Rego et al. 2006). However, no clear conclusion has been reached and the underlying genetic mechanisms of σ^2^p and FA have yet to be evaluated. In hybrids, the new genetic background often results in significantly increased FA, and therefore hybrid populations could be suitable for investigating the extent of congruence between DI and canalization.

Hybridization is a common phenomenon within the genus *Bacillus* Latreille, (Phasmatodea: Bacillidea) and has given rise to several hybrid parthenogenetic species (see Scali et al. 2003). *Bacillus rossius* spreads over most of the western Mediterranean basin with two Italian sub-species, *B. r. rossius* Rossi and *B. r. redtenbacheri* Nasceti & Bullini. *B. r. rossius* is found on the Thyrrenian and Ligurian coasts of the Italian mainland and on the North-Western part of Sardinia. *B. r. redtenbacheri* is found along the Adriatic coasts, on Sicily, and on the South-eastern part of Sardinia (Sarrabus area). A previous study of walking sticks, also called stick insects, on Sardinia revealed the presence of a hybrid zone between *B. r. rossius* and *B. r. redtenbacheri* based on allozyme analysis in the central part of the island (Mantovani and Scali 1991). But since that collection, the habitat of the stick insects has changed dramatically on Sardinia. Particularly along the coast, roads have been enlarged to improve the infrastructure for the increased number of tourists coming to the island, and the bush vegetation and distribution have become considerably fragmented. These changes have resulted in very heavy destruction of the habitats of the stick insects, because one of the preferred habitats of the stick insects is along roadsides where the usual feeding bush bramble (*Rubus fruticosus*) is very frequent. However, these changes have only affected the coastal areas of the island where the parental races live and not the centre where the hybrid zone presumably is found and can be considered undisturbed. In this study, stick insects from the presumed hybrid zone were used in order to investigate:

1) If the hybrid zone between *B. r. rossius* and *B. r. redtenbacheri* on Sardinia really exists based on recently developed microsatellite markers (Andersen et al. 2005).
2) The impact of hybridization on FA and σ^2^P in the naturally occurring hybrid zone between *B. r. rossius and B. r. redtenbacheri.*
3) The existence of congruence between σ^2^p and FA.

## Materials and Methods

### Collecting time and sites

Stick insects were collected from two nearby localities in the centre of Sardinia (Paulilatino and Escolca). These are the areas in which hybrid individuals between *B. r. rossius* and *B. r. redtenbacheri* have previously been found (Mantovani and Scali 1991). Due to habitat destruction, a sufficient number of amphigonic individuals of the parental *B. r. rossius* and *B. r. redtenbacheri* species on Sardinia that are found on the north eastern coast (Isola Rossa) and on the south-eastern coast (Villasimius), respectively, were not able to be obtained. In order to compare the hybrids with their parental races, amphigonic *B. r. redtenbacheri* were collected on the Adriatic coast of Italy at Torino di Sangro Marina, Abruzzo, and amphigonic *B. r. rossius* were collected on the Thyrrhenian coast at two nearby localities, Capalbio and Montiano, Toscana, which are about the same latitude as that of the *B. r. redtenbacheri* population and that of the hybrids. These populations of parental races were chosen because their habitats appeared very well preserved (see Pertoldi et al. 2001b) and because other samples taken at similar latitudes clearly clustered with the corresponding Sardinian *B. r. redtenbacheri* and *B. r. rossius* in the allozyme data sets well apart from the hybrids (Mantovani and Scab 1991).

### Morphological measurements

The adult stick insects were preserved in alcohol and measured for three bilateral traits (the antenna, the labial palpus, and the maxillary palpus) and three unilateral traits (mesonotum, metanotum, and abdomen). The bilateral traits were measured with the use of a binocular microscope with a digital filar eyepiece (Los Angeles Scientific Instrument Company, Inc., www.lasico.com). The unilateral traits were measured with a digital calliper to the nearest 0.1mm.

To estimate the measurement error for the bilateral traits, a sub-sample of 30 individuals, picked at random, were measured twice. The second set of measurements was made without reference to the first set. A two-way ANOVA was conducted to test for significance of FA relative to measurement error (the difference between the two independent measures of FA) (following Palmer and Strobeck 1986). To quantify possible errors associated with measuring body size (the sum of the mesonotum, metanotum, and abdomen), 20 individuals were taken at random, and each individual body size was measured ten times. The within individual coefficient of variation for each mean was taken as an estimate of the measurement error, adding Haldane's (1955) correction for small sample size.

The interaction mean square (MS) containing information about FA was tested against error MS (reflecting measurement error) showing that FA was significantly larger than measurement error for all three traits (p < 0.001). The measurement error for body size was low with a mean value of 0.12%.

### Genetic analysis

DNA was extracted from all 46 individuals collected at the presumed Sardinia hybrid zone, from 28 *B. r. rossius* individuals, and from 29 *B. r. redtenbacheri* individuals by the use of the CTAB method modified from Doyle and Doyle (1987). The stick insects were genotyped by five microsatellite loci (locus B154, B67, B101, B152, B198) (Andersen et al. 2005), and the fragment analyses of the PCR products were performed on a Beckman CEQ8000 sequencer using 5′labelled (Sigma-Proligo, www.sigmaaldrich.com) forward primers.

### Statistical analysis

In all of the following statistical analyses, males and females were analyzed separately, except in the genetic analysis.

The hybrids were collected from two nearby localities on Sardinia, and *B. r. rossius* individuals were collected from two nearby localities on the mainland. Before making any pooling of hybrid or *B. r. rossius* individuals from the two localities into one sample, the samples were analyzed for significant differences between localities with the respective tests described below. In no case were significant differences found between the collection sites within the two groups, and therefore the samples were pooled as one in all cases. Due to the large number of tests conducted, a sequential Bonferroni test (Rice 1989) was applied.

### Comparison of trait size among populations

Tests for significant changes in mean body size and mean length of the right side of the labial and maxillary palpus were performed by a one-way ANOVA and a Kruskal-Wallis test (Zar 1984). Multiple comparisons were done with Tukey's HSD test. A permutation *t*-test was performed among males and females in order to test for differences in the mean length of the right antenna between *B. r. rossius* and *B. r. redtenbacheri.* The hybrids were not included due to the high number of phenodeviants (see below).

### FA measurements

FA characterized by a normal distribution of right-left side differences with a mean of zero (Palmer and Strobeck 1986). To test whether the data obtained display the statistical properties of FA, a one sample *t*-test was performed for directional asymmetry, which occurs if the data set shows a significant deviation from zero of the mean value of right minus left trait size (r-1). Test for normality was performed with a Shapiro-Wilk test (Zar 1984).

FA of the three bilateral traits was calculated as (r-1) values, and for each population and sex, FA1 was estimated as the mean value of the absolute FA and FA_4_ as the variance of (r-1) (following Palmer and Strobeck 1986). Test for correlation between trait size and its absolute FA value was done by linear regression analysis and Spearman rank correlation test (Zar 1984), and correlations of absolute FA of different traits within individuals was tested by linear regression analysis.

### Comparison of FA and σ^2^p among populations

Due to the low sample size of the hybrids, pair-wise comparisons of FA1 were performed by a permutation *t*-test (1000 permutations). The permutation *t*-test is more accurate when dealing with non-normal distributions and small samples. Tests for differences in FA_4_ among populations were performed by an *F*-test.

The σ^2^P was estimated for the variance of body size (sum of the mesonotum, metanotum, and abdome*n*) and the variance of the right side of the three bilateral traits. Tests for differences in σ^2^P among populations were done by an *F*-test. Due to the small sample sizes, and since the *F*-test is rather sensitive to the assumption of normality, a Levene's (1960) test was also performed. This was done by transforming the data of body size or length of the right side of the bilateral traits into absolute deviations from the mean, and then testing for significant difference between the mean deviations by a permutation *t*-test (1000 permutations).

### Genetic analysis

The presence of null alleles was tested by the use of the software MicroChecker 2.2.3 (Oosterhout 2003). Observed (HO) and expected (H_E_) heterozygosities were calculated and tests for deviation from the Hardy-Weinberg equilibrium (HWE) and linkage disequilibrium (LD) were performed by the use of F_STAT_ 2.9.3.2 (Goudet 2002). Genetic differences between the populations were tested for by estimating the pair-wise F_ST_ value (Weir and Cockerham 1984) with Fstat 2.9.3.2 software (Goudet 2002). A Bayesian analysis was performed with STRUCTURE 2.0 software to infer the hybrid status of the Sardinian population (Pritchard et al. 2000) with a burn-in length of 100,000 and 1,000,000 Markov-chain Monte Carlo replicates for k = 3, where k represents the number of populations with distinctive allele frequencies. An admixture model incorporating predefined population information for the *B. r. rossius* and *B. r. redtenbacheri* populations was used, but no prior information was given for the hybrids. Incorporation of the population information for the pure species could be the most informative when dealing with hybrid zones (Prichard et al. 2000). Five independent simulations were compared to examine the consistency of estimated parameter values.

**Table 1.**
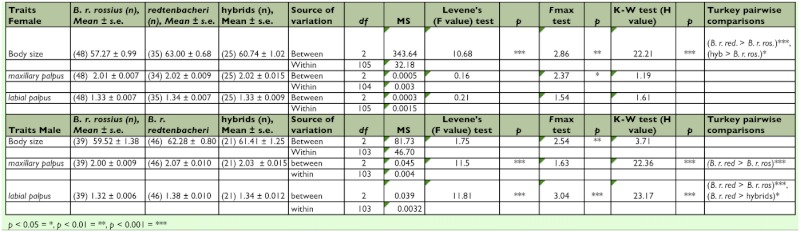
One-way ANOVA (Levens's test) for comparing the mean body size and mean length of the right *maxillary palpus* and *labial palpus in the parental B. rossius rossius and B. rossius redtenbacheri populations and their hybrids. F-test for homogeneity of variances, Kruskal-Wallis one-way non-parametric analysis and Tukey's pairwise comparisons.*

## Results

### Comparison of trait size among populations

For all traits and in both sexes, *B. r. redtenbacheri* showed the highest mean trait size (Table 1). Male hybrids always exhibited trait sizes intermediate between the parental populations (Table 1). Hybrid females also exhibited a mean body size that was intermediate between the parentals, but mean sizes of the labial and the maxillary palpus were equal to one of the parental populations (*B. r. rossius* and *B. r. redtenbacheri,* respectively) (Table 1).

Significantly higher mean body size was found in female *B. r. redtenbacheri* when compared to *B. r. rossius.* No significant differences in body size were observed between the male subspecies, but *B. r. redtenbacheri* exhibited a significantly higher mean maxillary palpus size than *B. r. rossius,* and for the labial palpus a significant higher mean size when compared to both *B. r. rossius* and the hybrids.

For males, the permutation *t*-test revealed a significantly higher mean length of the antenna in *B. r. redtenbacheri* than in *B. r. rossius* (*t*45,38 = 5.39, p < 0.001). This test did not consider the hybrids because of the high number of phenodeviants for this trait (see below). No significant difference in mean size of the antenna was observed between the females.

### FA measurements

Deviations from a mean of zero for the FA distributions were found for the antenna of the *B. r. redtenbacheri* females (*t* = 2.83, p < 0.01, df = 34), *B. r. rossius* females (*t* = 2.98, p < 0.01, df = 47), and *B. r. rossius* males (*t* = 2.90, p < 0.01, df = 38). Significant deviations from normality were found due to leptokurtic distributions of FA, but only in three cases: labial palpus and maxillary palpus in *B. r. redtenbacheri* males and antenna in hybrid males.

In no case was a significant correlation between absolute FA and body size found. However, correlations between absolute FA of different traits within individuals were found in several cases, but only in the parental subspecies. Significant correlation between absolute antenna FA and maxillary palpus FA was found in *B. r. rossius* females (*n* = 48, r = 0.53, p < 0.0001), *B. r. redtenbacheri* females (*n* = 35, r = 0.68, p < 0.0001), and *B. r. redtenbacheri* males (*n* = 45, r = 0.97, p <0.0001). Significant correlation between FA of the antenna and FA of the labial palpus was only found in *B. r. redtenbacheri* males (*n* = 45, r = 0.94, p < 0.0001). Significant correlation between absolute FA of the maxillary palpus and the labial palpus were found in *B. r. redtenbacheri* (*n* = 45, r = 0.96, p < 0.0001) and in *B. r. rossius* males (*n* = 39, r = 0.42, p < 0.01).

**Table 2.**
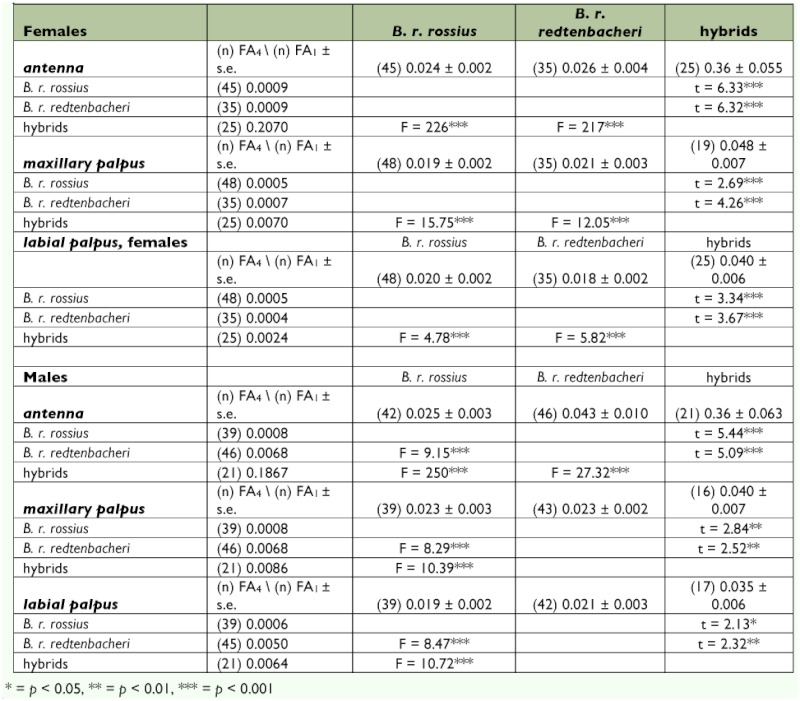
*t*-test for pair-wise comparisons of FA1 among the parental populations *B. rossius rossius and B. rossius redtenbacheri and their hybrids, and F-test for pairwise comparisons of FA4 among the parental B. rossius rossius and B. rossius redetnbacheri and their hybrids.*

### Comparison of FA and σ^2^p among populations

A very high number of phenodeviants for the antenna were found among the hybrid individuals of both sexes. A phenodeviant was defined in this study as an individual showing strong asymmetry exceeding three standard deviations of the signed values of FA due to a reduction in segments of one of the two antennae (45.65% phenodeviants among the hybrids versus 3.75% phenodeviants among the parentals), which caused the high values of FA for the hybrids. For both sexes, the permutation *t*-test found significantly higher levels of FA1 for all traits in the hybrids when compared to the *B. r. rossius* and *B. r. redtenbacheri* populations (Table 2). FA_4_ was significantly higher for all traits in the hybrid females when compared to the females from both parental races (Table 1). No significant differences in FA_4_ among *B. r. redtenbacheri* and *B. r. rossius* females were ever found (Table 1). A different pattern, however, was observed for the males where significant higher FA_4_ was found in both the hybrids and in *B. r. redtenbacheri* when compared to *B. r. rossius* males for all three traits (Table 2). Only for the antenna did the hybrid males show higher levels of FA_4_ when compared to both the parental subspecies due to the high number of phenodeviants among the hybrids (Table 1).

The *F*-test and Levene's (1960) pair-wise comparisons of σ^2^p gave the same results (only results of Levene's test are shown in the text). For body size, a significant higher σ^2^p in *B. r. redtenbacheri* was found when compared with *B. r. rossius* for both females (*t*35,48= 2.98, p < 0.01) and males (*t*39,46= 3.62, p < 0.01). No differences in σ^2^p were found for the bilateral traits, except from higher σ^2^p of the antenna of the hybrids when compared to both parental subspecies due to the high number of phenodeviants.

**Table 3.**
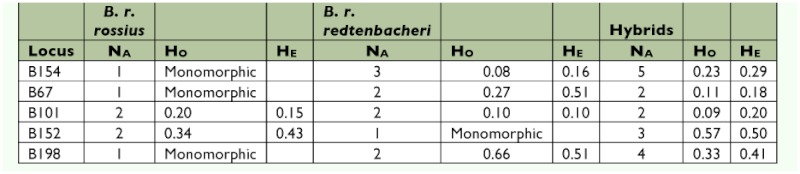
Number of observed alleles per locus (NA), observed frequency of heterozygotes (Ho) and expected frequency of heterozygotes (HE) for a population.

### Genetic analysis

No evidence for the presence of null alleles was found in the *B. r. rossius* individuals; however, in the hybrids evidence for the presence of null alleles at locus B101 (with a frequency of 0.18) and in the *B. r. redtenbacheri* individuals at locus B67 (with a frequency of 0.21) was found. In the *B. r. rossius* individuals, three loci were monomorphic, while Ho for the other two loci was 0.2 and 0.34 (Table 3). Two loci were monomorphic in the *B. r. redtenbacheri* individuals, and in the other loci H_O_ varied from 0.08 to 0.66 (Table 3). In the hybrids, no loci were monomophic and the observed heterozygosity varied from 0.11 to 0.57 (Table 3). No deviation from HWE was found in any of the populations, and no LD was found between any loci in the populations.

The estimator of population subdivision F_ST_ (Weir and Cockerham 1984) significantly differed from zero in all cases. Highest F_ST_ was found between the *B. r. rossius* and *B. r. redtenbacheri* populations with a value of 0.55, whereas the divergence between hybrids and the *B. r. rossius* or *B. r. redtenbacheri* populations was 0.11 and 0.33, respectively.

**Figure 1.**
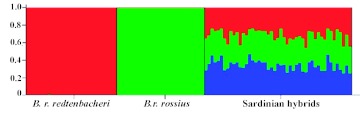
Bar plotting of the results obtained from the STRUCTURE analysis using *K*= 3. Each individual is represented as a vertical line partitioned into *K* shaded segments that represent an individual's estimated membership fraction in each of the *K* inferred clusters. High quality figures are available online.

The Bayesian assignment test resulted in high posterior probabilities (0.99) for the assignment of individuals from the parental subspecies to either *B. r. rossius* or *B. r. redtenbacheri* populations. For the hybrids, the situation was quite different with posterior probabilities of assignments of 0.32, 0.34, and 0.34 to the *B. r. rossius, B. r. redtenbacheri,* and the Sardinian population, respectively (Figure 1).

## Discussion

The collection on Sardinia clearly showed that the amphigonic populations of the two parental species have declined considerably in the coastal areas since the last collection by Mantovani and Scab (1991). The population present in the centre of the island still consisted of hybrids between *B. r. rossius* and *B. r. redtenbacheri.* This was confirmed both by our morphologic and genetic analysis. Morphologically, the trait sizes of the hybrids were found to be intermediate when compared to the parental subspecies (Table 1).

Genetically, the hybrids diverged significantly from both parental populations (estimated as FST values), and the Bayesian assignment test found no evidence of purely parental individuals in the hybrid zone (Figure 1). The hybrid status of the population found in the centre of Sardinia was further demonstrated by the assignment of the individuals with a probability of 33% to each of the parental populations and 33% probability to the Sardinian population (Figure 1). The microsatellite loci used in this investigation are not very polymorphic and would perform weakly in intraspecies population analyses, but due to the presence of subspecies specific alleles they were extremely useful for detecting hybrids.

The second aim of this investigation was to evaluate DI of the hybrids when compared to their parental subspecies. DI estimated as FA1 was significantly increased in the hybrid males and females when compared to both the parental subspecies (Table 2). The increased FA1 in the hybrids was attributed to genetic incompatibilities having a strong negative effect on development.

In females, FA_4_ exhibited the same pattern as FA1. However, a significantly higher FA_4_ was found in both the hybrids and *B. rossius redtenbacehri* males when compared to the *B. r. rossius* males. The increased FA_4_ in the hybrid males likely is caused by genetic incompatibilities, though the reasons for the higher FA_4_ in *B. r. redtenbacheri* males when compared to *B. r. rossius* males remain unclear. It is unlikely that environmental factors are responsible for the increased FA_4_ in *B. r. redtenbacheri* males since males and females were collected in the same environment, and no increase in FA_4_ in *B. r. redtenbacheri* females was observed when compared to *B. r. rossius* females. Some unknown genetic or genotype environment interactions could cause the increased FA_4_ in *B. r. redtenbacheri* males.

Among the hybrid individuals a very high number of phenodeviants for the antenna trait were found. It has previously been suggested that the genes controlling the antenna are located on the sex chromosomes (Andersen et al. 2005; Pertoldi et al. 2001b), and this investigation further supports this view. The genes located on the sex chromosomes in stick insect males are subject to strong selection because males are hemizygous for X-linked genes. For this reason, in hybrids, traits controlled by genes located on the X-chromosomes may be more affected by genetic incompatibilities than autosomal traits.

In this investigation the increase of FA in the hybrids was not correlated with an increase in (σ^2^p, even though σ^2^p often has been found to increase due to stress, both environmental and genetic (Imasheva and Bubliy 2003; Willmore et al. 2005, 2006). There is good reason to believe that in the *B. rossius* race hybrids, genetic variability (σ^2^g) has increased because of their higher allele diversity when compared to the parental subspecies. This was also in concordance with a previous study of allozymes in the hybrids (Mantovani and Scab 1991). Nonetheless, this was not paralleled by an increased σ^2^p in the hybrids. Therefore the environmental variability (σ^2^e) could be increased in the parental subspecies, counteracting the increased σ^2^g in the hybrids, and resulting in the same level of σ^2^P. A cooccurring increase of σ^2^P in the parental populations seems unlikely, however, since it should have occurred in all collection sites which are apparently undisturbed and located far apart on the Tyrrhenian and Adriatic coasts.

If σ^2^P was increased in the parental populations, suggesting that the hybrids have developed in a more stable environment, it would actually reinforce the hypothesis that the increased FA observed in the hybrids was actually due to the new genetic structure caused by hybridization. More likely the lack of a concordant increase of both σ^2^p and FA in the hybrids could be explained by the hypothesis suggested by Waddington (1957), that separate mechanisms are responsible for the impact of stress (in this case “genetic stress” caused by hybridization) on σ^2^p and FA: σ^2^p would be the result of canalization processes, whereas FA would be the result of DI.

Following the Bateson-Dobzhansky-Muller (BDM) model, genetic incompatibilities between species arise when, in one species, a new allele at a given locus becomes fixed, and in the other species a second new allele at another locus becomes fixed (Orr 1995; Gavrilets 1999). Lynch and Force (2000) have made a further elaboration on this model that suggests that duplicated genes, which are present in high numbers in almost all eukaryotic organisms, are the substrate for the origin of genomic incompatibilities in isolated populations. Genetic incompatibilities arise when one population loses function from one gene copy while the second loses function from a second gene copy at a different chromosomal location, which leads to chromosomal re-pattering such that gametes produced by the hybrid individuals can be completely lacking functional genes for a duplicate pair (see Lynch and Force 2000 for details). Duplicate gene mutations leading to genetic incompatibilities can accumulate in an effectively neutral manner (Lynch and Force 2000). This is opposite to co-adapted gene complexes that are believed to arise due to selection acting to preserve non-additive, epistatic interactions among loci. This creates interacting complexes of genes adapted to the local environmental conditions (Parsons 1990).

If σ^2^p is mostly controlled by canalization, hybridization between individuals with different co-adapted gene complexes would lead to disruption of epistatic genetic interactions. Then σ^2^p would increase due to the lack of epistatic modifiers that suppress the additive genetic variance and the environmental variance under genetic and environmental canalization, respectively (Wagner et al. 1997). However, if the genetic differences between species are mainly due to genetic incompatibilities that have arisen in a neutral/passive manner, as suggested by Lynch and Force (2000), FA could increase due to divergence between species in regulatory pathways, resulting in high levels of asymmetry which do not need to increase (σ^2^p as well.

If the observed increase in FA is due to increased DI, this depends on the definition of DI. Rego et al. (2006) define developmental stability (which is the opposite of DI) to be the buffering mechanism against random noise in the cellular processes that are involved in the development of morphological structures. If this definition holds, the observed increased FA in interspecific hybrids is rarely the result of randomness; it is rather the result of well-defined genetic incompatibilities and therefore does not reflect DI. If DI, on the other hand is defined as the inability of an organism to buffer its developmental processes against environmental and genetic disturbances to ensure bilateral symmetry, the observed increase in FA in this study is the result of increased DI in the hybrids. No matter whether the increased FA in this study reflects increased DI or not, it can be concluded that the mechanisms responsible for the increased FA in the hybrids do not seem to affect the buffering mechanism responsible for σ^2^p.

This study clearly shows that the hybrids are more asymmetric than their parents. The study found no reason to assume that the increase in FA is due to environmental factors, and therefore the increased FA can be attributed to genetic incompatibilities in the hybrids. The reason for no concordant increase in σ^2^p in the hybrids has been debated. However, due to the incomplete knowledge of the genetic mechanisms behind FA and σ^2^p, a lot of work must be done in order to clarify how the two processes responsible for intra- and inter-individual variation are controlled and interact. Hopefully, these results will promote additional investigations on the topic.

## Abbreviations

DI: developmental instability;
FA: fluctuating asymmetry;
σ^2^p: phenotypic variability
